# *In silico* exploration of deep-sea fungal metabolites as inhibitor of Ebola and Marburg VP35 and VP40

**DOI:** 10.1371/journal.pone.0307579

**Published:** 2024-07-25

**Authors:** Abdullah R. Alanzi, Mohammed F. Alajmi, Mohammed S. Al-Dosari, Mohammad K. Parvez, Moneerah J. Alqahtani

**Affiliations:** Department of Pharmacognosy, College of Pharmacy, King Saud University, Riyadh, Saudi Arabia; Cholistan University of Veterinary and Animal Sciences, PAKISTAN

## Abstract

VP30 and VP40 proteins of Ebola and Marburg viruses have been recognized as potential targets for antiviral drug development due to their essential roles in the viral lifecycle. Targeting these proteins could disrupt key stages of the viral replication process, inhibiting the viruses’ ability to propagate and cause disease. The current study aims to perform molecular docking and virtual screening on deep-sea fungal metabolites targeting Marburg virus VP40 Dimer, matrix protein VP40 from Ebola virus Sudan, Ebola VP35 Interferon Inhibitory Domain, and VP35 from Marburg virus. The top ten compounds for each protein target were chosen using the glide score. All the compounds obtained indicate a positive binding interaction. Furthermore, AdmetSAR was utilized to investigate the pharmacokinetics of the inhibitors chosen. Gliotoxin was used as a ligand with Marburg virus VP40 Dimer, Austinol with matrix protein VP40 from Ebola virus Sudan, Ozazino-cyclo-(2,3-dihydroxyl-trp-tyr) with Ebola VP35 Interferon Inhibitory Domain, and Dehydroaustinol with VP35 from Marburg virus. MD modeling and MMPBSA studies were used to provide a better understanding of binding behaviors. Pre-clinical experiments can assist validate our *in-silico* studies and assess whether the molecule can be employed as an anti-viral drug.

## Introduction

The proteins VP30 and VP40 are identified in the Filoviridae family’s Ebola and Marburg viruses. These viruses cause severe and frequently fatal hemorrhagic fever in humans and other primates. Both VP30 and VP40 are structural proteins that play key functions in the viral lifecycle [1].

VP30 is a protein that activates transcription. It functions as a cofactor for the viral RNA-dependent RNA polymerase (RdRp) complex, which is required for viral genome transcription and the production of new viral RNA molecules. VP30 is involved in the regulation of the transition between viral gene transcription and replication. This protein is essential for maintaining the equilibrium between viral gene replication and production of viral RNA and proteins [2–4].

VP40 is a matrix protein with several activities during the viral lifecycle. It is involved in the assembly and release of freshly generated viral particles. VP40 interacts with viral RNA and other structural proteins to form the viral nucleocapsid, which is the virus’s central core and contains the viral RNA genome. Furthermore, VP40 is required for viral budding from the host cell’s plasma membrane. It drives the membrane curvature required for the development of the viral envelope and enables the release of mature viral particles from the infected cell [5–7].

Both VP30 and VP40 are required for the replication and propagation of the Ebola and Marburg viruses. Because of their importance in the viral lifecycle, they are potential targets for antiviral drugs and vaccine research [8].

Nature has long been recognized as the primary source of unique and novel bioactive compounds that aid in the treatment of many diseases and infections [9, 10]. Deep-sea fungal metabolites are bioactive substances produced by fungi living in deep-sea environments characterized by high pressure, low temperatures, and harsh circumstances. Researchers have become interested in these metabolites due to their potential for medicinal, biotechnological, and industrial applications. They are of interest due to their distinct chemical structures and potential bioactivities, which include antibacterial and antiviral capabilities. Some deep-sea fungus metabolites have showed potential in preventing viral proliferation [11, 12]. These chemicals could be investigated for their capacity to target viral proteins such as VP30 and VP40 in Ebola and Marburg viruses. Specific research on their activity against these proteins, however, would be required to confirm their efficacy. Hence, this study was designed to test deep sea fungal metabolites against VP30 and VP40 of Ebola and Marburg virus. The study of their interactions with the target proteins may help in the development of novel drugs.

## Methodology

### Protein and ligand structures retrieval

The protein structures of Marburg virus VP40 Dimer, matrix protein VP40 from Ebola virus Sudan, Ebola VP35 Interferon Inhibitory Domain, and VP35 from Marburg virus with PDB IDs 5B0V, 3TCQ, 3FKE, and 5TOI respectively were retrieved from Protein Data Bank [13]. Modeller v9.22 was used for checking for the missing residues and charges that were then repaired accordingly [14]. Structure visualization was performed in PyMOL [15]. PubChem database was used to gather the structural and functional properties of compounds; Gliotoxin, Austinol, Ozazino-cyclo-(2,3-dihydroxyl-trp-tyr), and Dehydroaustinol [16].

### Virtual screening

Significant inhibitors or lead compounds can be identified from a vast chemical pool using a technique called structure-based virtual screening, or SBVS. For this computational technique to work, interactions within the target proteins’ active or binding pockets are critical. Using MCULE, a web-based library with millions of possibly synthesized compounds, helped finish SBVS [17].

The structural information of the tertiary proteins was submitted as PDB files. Molecular mass (Mw) less than 500 Daltons hydrogen, bond donor (HBD) and acceptor (HBA) parameters less than 5, number of rotatable bonds less than 10, and log P (octanol-water partition coefficient) parameter less than 5 was chosen as the filtration parameters [18]. A sample size of 1000, a similarity criterion of 0.70, and a cap of 3 million compounds after sphere exclusion were also chosen as parameters for selection. Those three variables were the only changes made to the MCULE settings. In this study, more than 5,000,000 different ligands were used to examine the CMAH active site. The molecular docking of CMAH with the naturally occurring ligand was performed using the specified grid box. Vina integration was employed in the software for the digital screening. The two strongest inhibitors were chosen after the Vina-docking score was used to evaluate the binding affinities of the inhibitors. Additionally, AdmetSAR was used to study the pharmacokinetics of the various selected inhibitors. The physical characteristics of the target protein and the amino acid residues that interact with it were studied. The study is based on the target protein’s physical characteristics and the interacting amino acid residues.

### Molecular dynamics simulation

Molecular dynamic simulation is a computational technique for validating docking findings and gaining insight into the atomic or molecular characteristics of biological macromolecules. In this study, the MD simulation of following four protein-ligand complexes: Marburg virus VP40 Dimer _Gliotoxin, matrix protein VP40 from Ebola virus Sudan_Austinol, Ebola VP35 Interferon Inhibitory Domain_Ozazino-cyclo-(2,3-dihydroxyl-trp-tyr), and VP35 from Marburg virus_ Dehydroaustinol was carried out using GROMACS [19] in Linux environment for 100ns. The protein-ligand complex was solvated in a water box containing TIP3P water molecules along with extra sodium and chloride ions to mimic the conditions found in the body after the ligand had been extracted and optimized with the aid of AutoDockTools [20]. After a 200ps energy-efficient steepest descent, the system was given time to reach thermodynamic equilibrium with the aid of the CHARMM36 force field and GROMACS, and only then was a 100ns production MD simulation performed [21]. Eventually, programming languages, Python, PyMOL, and VMD, together with the gmx rms, gmx RMSF, gmx area, and gmx cod and gyrate tools [15] were used for analysis of the output files and analysis result of Root mean square deviation (RMSD), Root mean square fluctuation (RMSF), Radius of gyration (Rg), Solvent accessible surface area (SASA), and number of hydrogen bonds was plotted in the form of scatter line graphs.

### Binding free energy calculation by MMPBSA

The binding free energies of the complexes were calculated by employing the MMPBSA method. The binding free energies were determined by subtracting the receptor and ligand free energy from the complex free energy (Eq 1). Further, the entropy changes were calculated by employing Eq 2. Moreover, the residue binding energy decomposition was estimated to find the key interacting residues. Eq 3 was used for the calculation of binding free energy decomposition.


$$\Delta G_{Bind}=\Delta G_{complex}‐(\Delta G_{protein}+\Delta G_{ligand})$$ (1)

$$T\Delta G=T(\Delta S_{trans}+\Delta S_{rot}+\Delta S_{vib})$$ (2)

$$\Delta G_{Inhibitor‐residue}=\Delta G_{vdW}+\Delta G_{ele}+\Delta G_{ele,\;sol}+\Delta G_{nonpol,\;sol}$$ (3)

## Results

### Selection of best inhibitor for proteins

The compounds’ structures were obtained from PubChem and virtually screened according to their physicochemical characteristics. The top 10 compounds for each protein target (Marburg virus VP40 Dimer, matrix protein VP40 from Ebola virus Sudan, Ebola VP35 Interferon Inhibitory Domain, and VP35 from Marburg virus) were chosen. The chosen chemicals underwent a pharmacokinetics analysis. Table 1 lists the targeted proteins’ chosen inhibitors. The docking scores of the selected compounds against the respective proteins are shown in Table 2.

**Table 1:** Selection of best inhibitor for protein based on physiochemical properties of protein-ligand complexes.

No.	Compounds	MW (g/mol)	HBD	HBA	LogP	nRot	RO5 (Violations)
**Ebola VP35 Interferon Inhibitory Domain**							
**1**	**Ozazino-cyclo-(2,3-dihydroxyl-trp-tyr)**	**365.1**	**3**	**7**	**4.56**	**2**	**1**
**2**	Circumdatin F	291.1	1	5	1.79	0	0
**3**	Cochliodone A	638.2	0	12	3.09	4	2
**4**	Austin	500.2	1	9	2.50	2	2
**5**	Dicitrinone B	438.2	4	6	5.15	2	1
**6**	Lithocarin A	430.2	2	5	2.76	8	0
**7**	Brevione B	424.2	0	4	4.33	0	0
**8**	Verlamelin B	871.5	9	18	1.95	15	4
**9**	Penicitrinone F	394.1	2	5	6.29	0	1
**10**	Ergosterol	396.3	1	1	6.47	4	1
**Matrix protein VP40 from Ebola virus Sudan**							
**11**	Brevione A	422.2	4	0	4.43	0	0
**12**	Brevione K	434.2	5	0	3.37	0	1
**13**	Brevione B	424.2	4	0	4.33	0	0
**14**	**Austinol**	**458.1**	**8**	**2**	**2.15**	**0**	**1**
**15**	Brevione J	440.2	5	1	3.39	0	1
**16**	Dehydroaustin	498.1	9	0	2.55	2	1
**17**	Chrysamide B	524.1	12	0	4.10	8	2
**18**	Clavatustide A	471.1	8	2	3.48	3	1
**19**	Luteoalbusin A	464.1	7	3	3.46	2	1
**20**	Penicitol D	428.1	7	3	5.18	2	2
**Marburg virus VP40 Dimer**							
**21**	Kojic acid	464.1	3	7	3.58	1	1
**22**	**Gliotoxin**	**336.1**	**1**	**6**	**1.81**	**3**	**0**
**23**	Physcione	464.1	3	7	3.46	2	1
**24**	o-Xylene-3,alpha,alpha’-triol	431.1	1	9	2.97	5	1
**25**	Terphenyllin	360.2	1	4	3.68	3	0
**26**	Cyclopenol	310.1	2	6	2.49	1	0
**27**	Versicolorin B	294.1	1	5	2.76	1	0
**28**	Butyrolactone i	304.0	1	6	1.39	0	0
**29**	Wentilactone A	326.1	2	5	4.34	1	0
**30**	Macrophorin A	338.1	3	5	2.31	1	0
**VP35 from Marburg virus**							
**31**	Verlamelin	885.5	9	18	2.28	15	4
**32**	**Dehydroaustinol**	**456.1**	**1**	**8**	**2.32**	**0**	**1**
**33**	Clavatustide B	457.1	2	8	3.14	2	1
**34**	Brevione K	434.2	0	5	3.37	0	0
**35**	Dehydroaustin	498.1	0	9	2.55	2	1
**36**	Brevione B	424.2	0	4	4.33	0	0
**37**	Varioxepine A	463.2	0	8	4	3	1
**38**	Brevione A	422.2	0	4	4.43	0	0
**39**	Brevione G	436.2	1	5	3.23	0	0
**40**	7-Hydroxydehydroaustin	514.1	1	10	2.00	2	2

**Table 2:** Glide score of protein-ligand complexes.

No.	Compounds	PubChem IDs	Glide score (kcal/mol)
**Ebola VP35 Interferon Inhibitory Domain**			
**1**	**Ozazino-cyclo-(2,3-dihydroxyl-trp-tyr)**	146115843	**-7.7**
**2**	Circumdatin F	10708688	-7.3
**3**	Cochliodone A	102516358	-7.6
**4**	Austin	38353601	-7.4
**5**	Dicitrinone B	50924225	-7.4
**6**	Lithocarin A	139590729	-7.4
**7**	Brevione B	101334419	-7.3
**8**	Verlamelin B	139588455	-7.3
**9**	Penicitrinone F	156581468	-7.3
**10**	Ergosterol	444679	-7.2
**11**	Licochalcone A*	5318998	-7.4
**12**	18β-Glycyrrhetinic Acid*	10114	-9.1
**Matrix protein VP40 from Ebola virus Sudan**			
**13**	Brevione A	139587535	-11.2
**14**	Brevione K	139587992	-11.1
**15**	Brevione B	101334419	-10.9
**16**	**Austinol**	3085683	**-10.9**
**17**	Brevione J	139583308	-10.9
**18**	Dehydroaustin	122201239	-10.4
**19**	Chrysamide B	132515915	-10.4
**20**	Clavatustide A	139587634	-10.4
**21**	Luteoalbusin A	71497282	-10.3
**22**	Penicitol D	146682883	-10.2
**23**	Licochalcone A*	5318998	-7.6
**24**	18β-Glycyrrhetinic Acid*	10114	-7.6
**Marburg virus VP40 Dimer**			
**25**	Kojic acid	3840	-9.7
**26**	**Gliotoxin**	6223	**-9.5**
**27**	Physcione	10639	-9.4
**28**	o-Xylene-3,alpha,alpha’-triol	81835	-9.4
**29**	Terphenyllin	100437	-9.3
**30**	Cyclopenol	16681741	-9.2
**31**	Versicolorin B	107849	-9.1
**32**	Butyrolactone i	123740	-9
**33**	Wentilactone A	156679	-9
**34**	Macrophorin A	158854	-9
**35**	Licochalcone A*	5318998	-8.6
**36**	18β-Glycyrrhetinic Acid*	10114	-9.6
**VP35 from Marburg virus**			
**37**	Verlamelin	139588823	-9.0
**38**	**Dehydroaustinol**	25235987	**-8.7**
**39**	Clavatustide B	139583980	-8.6
**40**	Brevione K	139587992	-8.5
**41**	Dehydroaustin	122201239	-8.4
**42**	Brevione B	101334419	-8.3
**43**	Varioxepine A	102366731	-8.3
**44**	Brevione A	139587535	-8.2
**45**	Brevione G	44139897	-8.2
**46**	7-Hydroxydehydroaustin	139583268	-8.2
**47**	Licochalcone A*	5318998	-8.2
**48**	18β-Glycyrrhetinic Acid*	10114	-8.2

(* control drugs for target proteins)

### Docking, MD simulation and MMPBSA of all protein-ligand complexes

CB Dock was used to carry out protein-ligand docking and then Discovery Studio was used to display the 3D structure of the docked complex and the residue-by-residue interactions between the docked molecules. Docked complexes were further evaluated through MD simulation and five factors were analyzed i.e., RMSD, RMSF, Radius of gyration, SASA, and number of hydrogen bonds in protein-ligand interaction.

A model system was used in which Gliotoxin was used as a ligand with Marburg virus VP40 Dimer, Austinol with matrix protein VP40 from Ebola virus Sudan, Ozazino-cyclo-(2,3-dihydroxyl-trp-tyr) with Ebola VP35 Interferon Inhibitory Domain, and Dehydroaustinol with VP35 from Marburg virus.

#### 1. VP35 from Marburg virus with Dehydroaustinol

**Fig 1:**
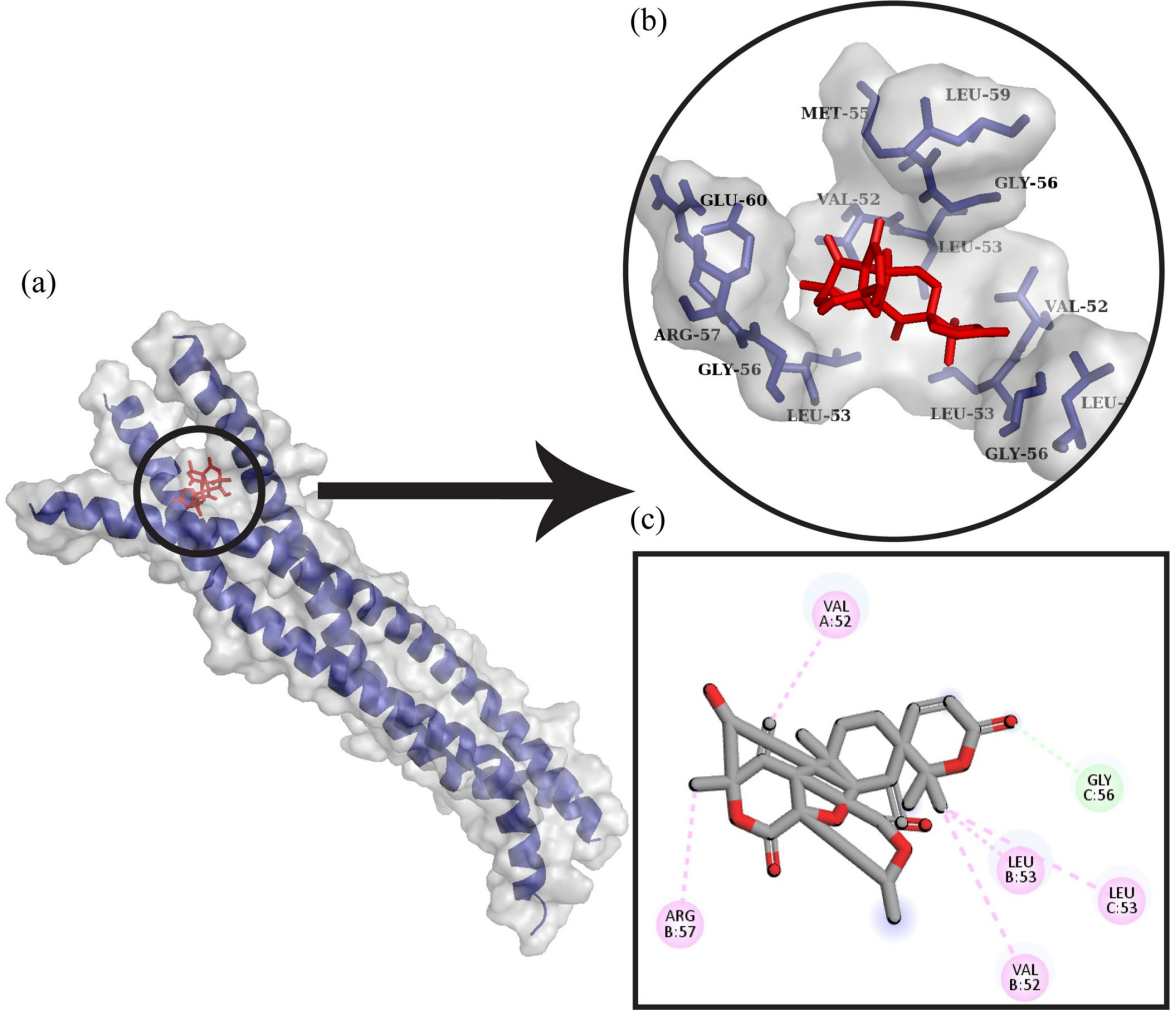
Protein-ligand docking. (a) The ligand Dehydroaustinol is docked with the protein VP35 from Marburg virus. (b) The interactive 3D viewer illustrating selected binding modes. (c) Residue wise interaction between ligand and protein obtained through Discovery Studio.

The docked complex of VP35 from Marburg virus and Dehydroaustinol is shown in Fig 1. It can be observed that Gly56 made a hydrogen bond with ligand while Val52A, Arg57B, Leu53B, Leu53C, and Val52B were involved in the hydrophobic interactions.

**Fig 2:**
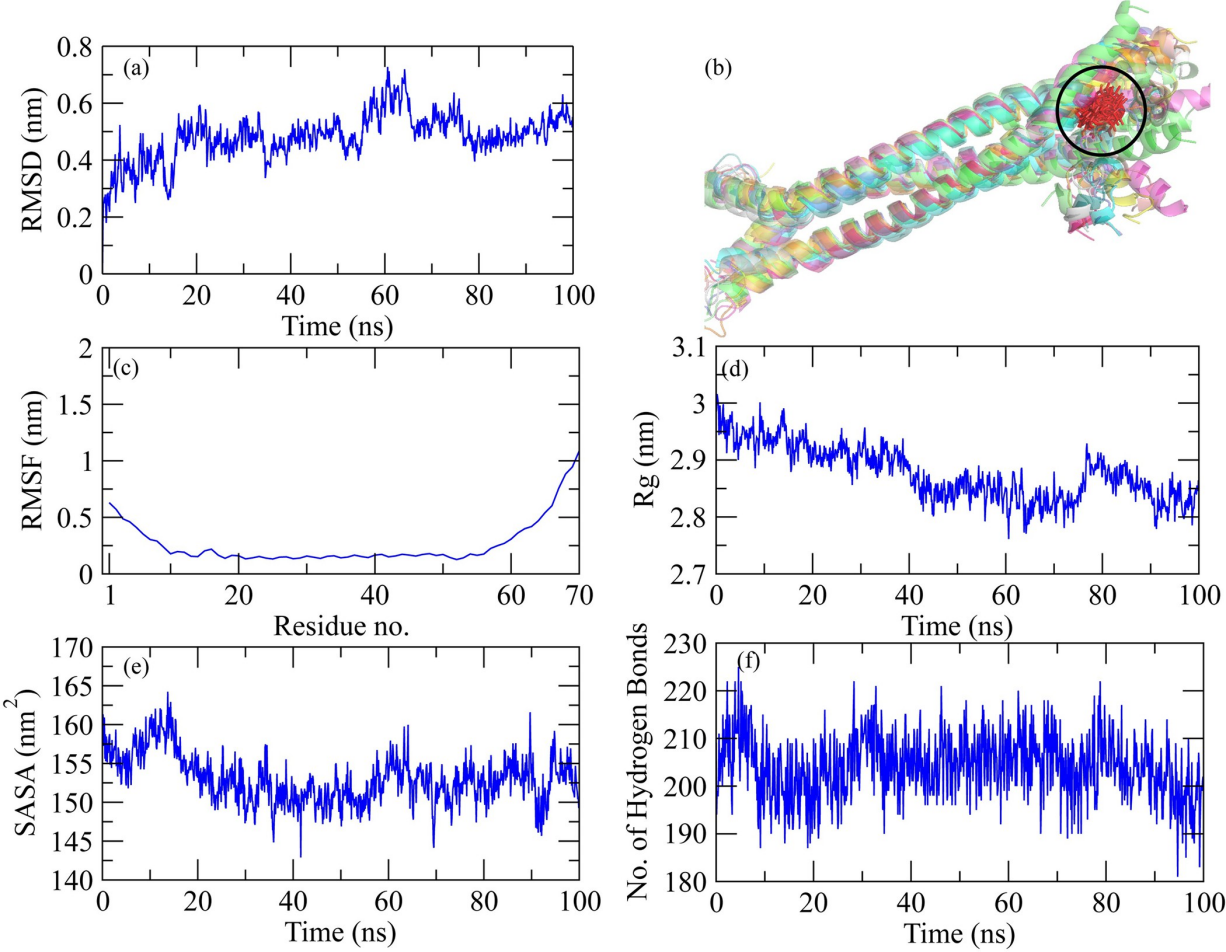
Graphical representation of molecular dynamic simulation at 100ns for VP35 from Marburg virus and Dehydroaustinol ligand. (a) RMSD for the stability analysis of the protein with ligand. (b) Alignment of the snapshots extracted from MD trajectory. (c) RMSF explains the flexibility of the protein as per the fluctuation of the amino acid. (d) The Radius of Gyration shows the compactness in the protein as the ligands bind to it. (e) The accessibility of the solvents for the protein can be predicted through the SASA value. (f) The breaking and formation of the hydrogen bonds with the passage of time is represented graphically.

**MD simulation of VP35 from Marburg virus with Dehydroaustinol.** The molecular dynamic simulation analysis for Ebola VP35 from Marburg virus and Dehydroaustinol protein-ligand complex is represented graphically. The RMSD of the protein after the binding of ligand is represented in Fig 2A. After the binding of ligand to the protein, it is observed that the RMSD value started increasing from the 0ns, which shows the less stable or weaker interaction between the protein and ligand over time. Further, the snapshots of protein-ligand complex were extracted at 0, 10, 20, 30, 40, 50, 60, 70, 80, 90, and 100 ns and then aligned to find the behavior of the complex during simulation (Fig 2B). It was observed that the ligand remained stably bound to the protein. The RMSF analysis is shown in Fig 2C. The graph shows the decrease in the RMSF value from the start which shows the decreased flexibility of protein after the binding of the ligand. The trend starts increasing at the end which shows the increase in flexibility of the protein-ligand complex. The decrease in the Rg value (Fig 2D) was observed as time passes after the binding of ligand to protein. The stability of the protein then changes from 78ns to 90ns as the Rg value for protein started increasing. SASA for the protein-ligand interaction is represented in Fig 2E. At the start, there was an increase in the SASA value till 15ns but then almost the trend started decreasing and it remained the same which shows that the accessibility of solvent to the protein decreases as the ligand binds to it. The number of hydrogen bonds (Fig 2F) explains the number of bonds that were formed or broken down during the protein-ligand interactions. The graphs showed similar trends for the number of hydrogen bonds of protein interaction with the ligand with the passage of time.

**Fig 3:**
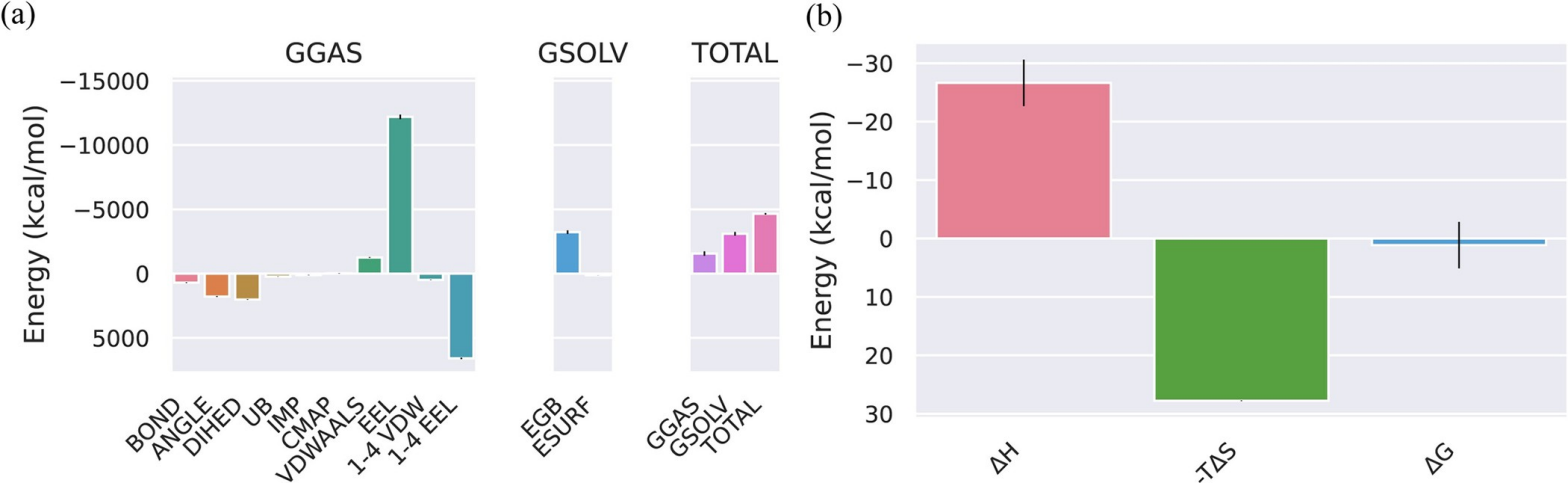
(a) The negative binding energy for the VP35 from Marburg virus_Dehydroaustinol complex represents the stability in the structure of the protein after the attachment of the ligand. (b) The decrease in the enthalpy makes the complex stable while the entropy shows an unfavorable interaction protein between the protein and the ligand.

**MMPBSA of VP35 from Marburg virus and Dehydroaustinol complex.** The different phases of change in the bonds and energy that takes place in it because of the binding of VP35 from Marburg virus with the ligand Dehydroaustinol are represented in Fig 3(A). The total energy of the protein and ligand complex decreases for both GGAS and GSOLV which shows the stability in the structure of the protein after the attachment of the ligand. The results of the binding energy of the following protein-ligand complex are represented in Fig 3(B). These results are computed from the GB and IE methods which are involved in the standard MM/GBSA calculations. A detailed analysis of the contributions showed the enthalpic component (ΔH) as favorable (negative value) to the binding process. At the same time, the entropic term (−TΔS) has given an unfavorable (positive value) of the energy.

#### 2. Matrix protein VP40 from Ebola virus Sudan_Austinol

**Fig 4:**
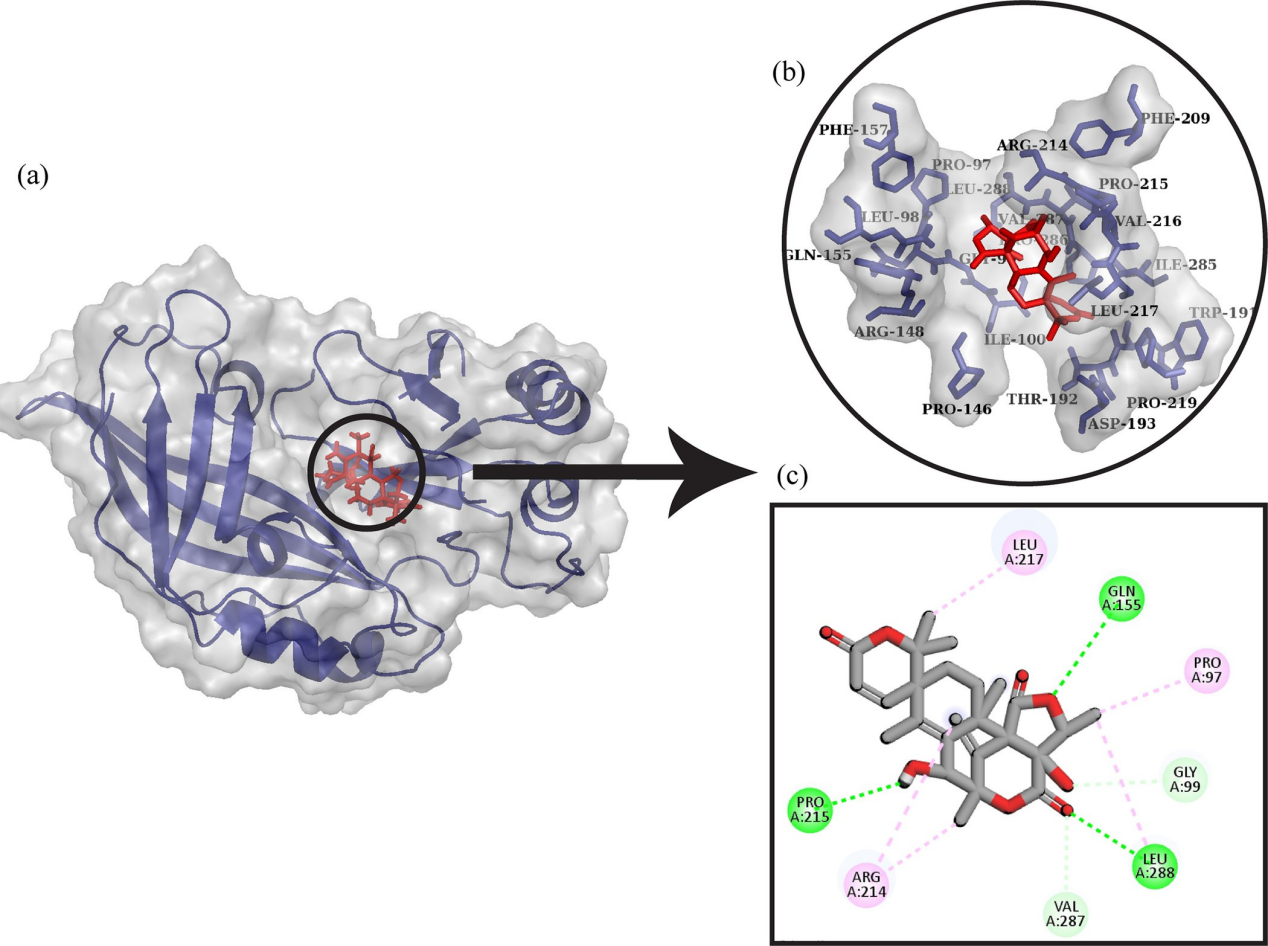
Protein-ligand docking. (a) The ligand Austinol is docked with Matrix protein VP40 from Ebola virus Sudan. (b) The interactive 3D viewer illustrating selected binding modes. (c) Residue wise interaction between ligand and protein obtained through Discovery Studio.

Matrix protein VP40 from Ebola virus Sudan and Austinol docked complex is shown in Fig 4. It can be observed that Austinol made five hydrogen bonds with Pro215, Leu288, Gln155, Gly99, and Val287. It also made alkyl interactions with Arg214, Leu217, and Pro97.

**Fig 5:**
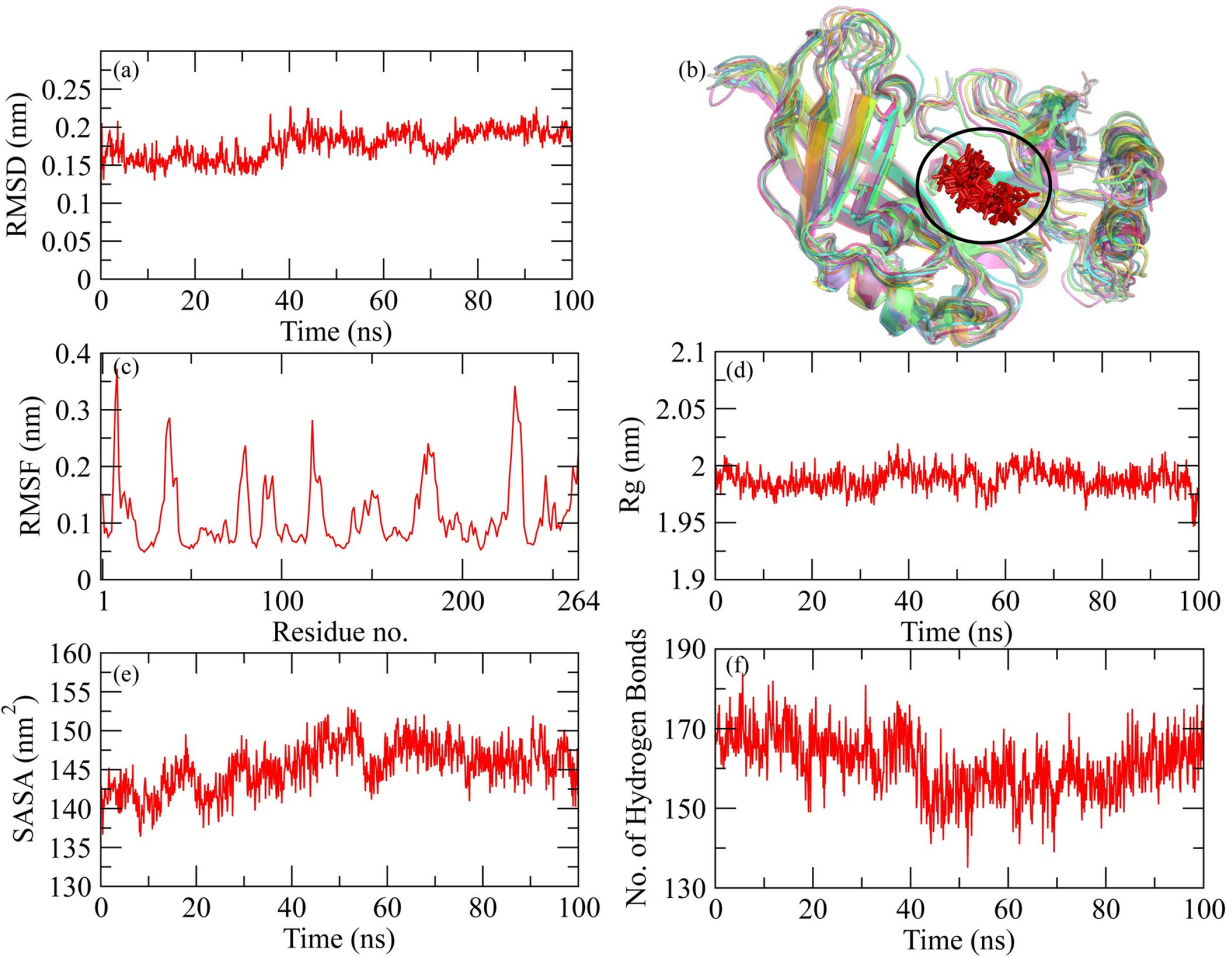
Graphical representation of molecular dynamic simulations of Matrix protein VP40 from Ebola virus Sudan with Austinol complex over 100ns. (a) RMSD graph showing the interaction between protein ligands. (b) Snapshots alignment (c) RMSF showing the fluctuation in protein ligand complex on different amino acid residues plotted on x-axis. (d) Radius of gyration showing the gain of compactness of complex towards the end of simulation. (e) SASA value indicating the stable protein ligand interaction. (f) Number of hydrogen bonds affecting the interactions of protein ligand complex.

**MD simulation of matrix protein VP40 from Ebola virus Sudan_Austinol.** RMSD analysis of Ebola virus Sudan matrix protein VP40 with Austinol is shown in Fig 5A. At the start of the simulation i.e., 0 ns and at 35 ns, there was increase in the RMSD value which indicates the decrease in the stability of Matrix protein VP40 interaction with Austinol and after 35 ns there was decrease in the RMSD value as compared to the start of the simulation resulting in an increase in stability. Similarly, the snapshots alignments showed that the ligand remained attached to the protein during simulation (Fig 5B). RMSF graph is displayed in Fig 5C. RMSF value was inconsistent throughout the simulation and there were almost 7 amino acid residues that showed the highest RMSF value and 0.37nm was the highest RMSF recorded at 9^th^ residue position. According to the Rg graph in Fig 5D, from 0ns to 36ns protein structure was stable upon interaction of Matrix protein VP40 with Austinol but after 36 ns structure became unstable because there was abrupt increase and decrease in the trend of Rg graph and at the end of simulation protein was more compact as compared to the initial point. According to the SASA graph in Fig 5E, from 0ns to 55 ns there was abrupt increase and decrease in the SASA value means conformational change in protein was dynamic but after 55 ns trend was smooth indicating that the interaction between protein and ligand was stable. Analysis of the number of hydrogen bonds in protein ligand complex is shown in Fig 5F. The highly fluctuating values show that several bonds were formed and broken every ns and no specific trend was followed overall.

**Fig 6:**
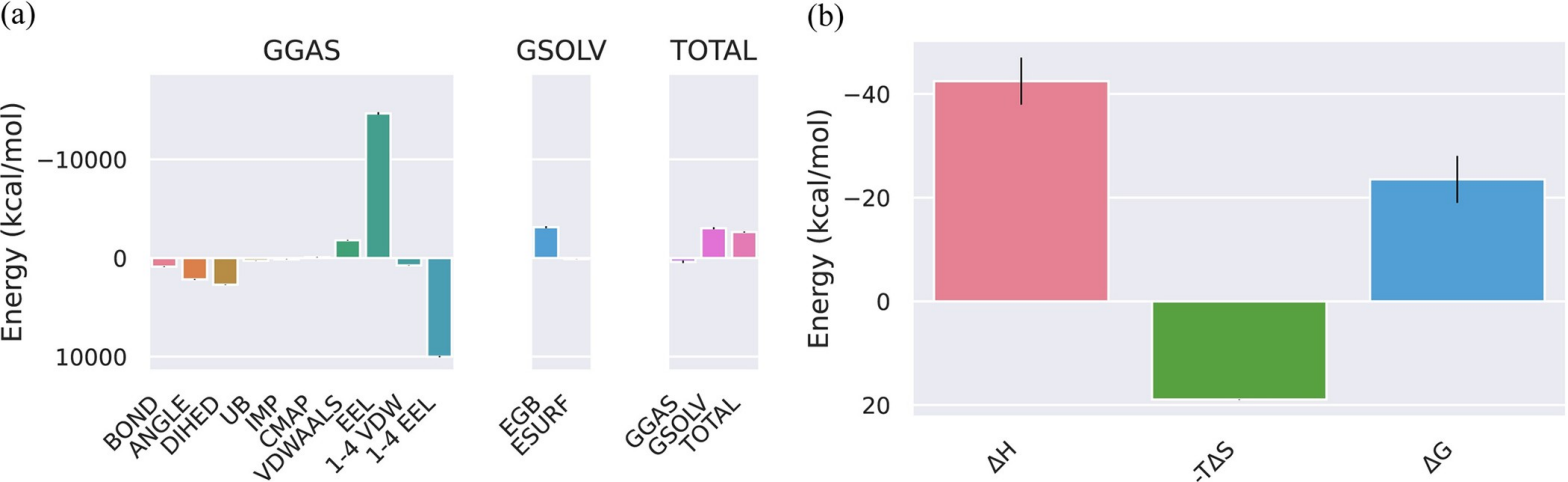
(a) The total energies of GGAS and GSOLV of the Matrix protein VP40_Austinol complex were calculated by the GB method. (b) The instability in the structure of the Matrix protein VP40_Austinol complex has been represented from the entropy values.

**MMPBSA of matrix protein VP40 from Ebola virus Sudan with Austinol.** The energetics of the Matrix protein VP40 to the ligand Austinol is represented in Fig 6. The different phases of Matrix protein VP40 and the change in the bonds and the energy that takes place in it because of the Matrix protein VP40 and Austinol as represented in Fig 6(A). The total energy of the protein and ligand complex decreases for both GGAS and GSOLV which shows the stability in the structure of the Matrix protein VP40 after the attachment of the ligand Austinol. The results of the binding energy of the Matrix protein VP40_Austinol complex are represented in Fig 6(B). These results are computed from the GB and IE methods which are involved in the standard MM/GBSA calculations. A detailed analysis showed the enthalpic component (ΔH) as favorable (negative value) to the binding process. At the same time, the entropic term (−TΔS) has given an unfavorable (positive value) of the energy. The Gibbs free energy (ΔG) has shown a decrease in the energy of the Matrix protein VP40_Austinol complex formation which explains the stability of the complex.

#### 3. Marburg virus VP40 dimer with Gliotoxin

**Fig 7:**
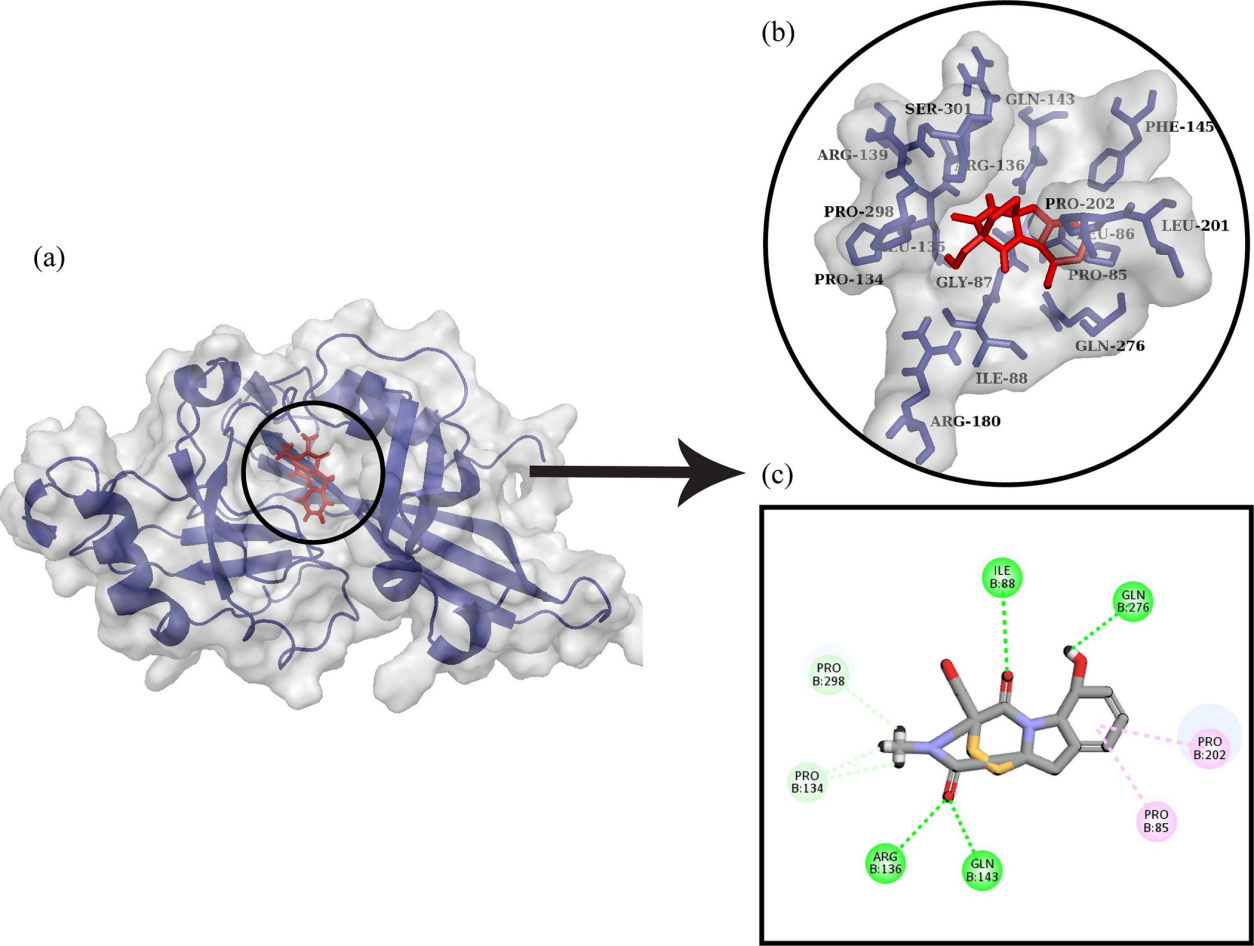
Protein-ligand docking. (a) The ligand Gliotoxin is docked with Marburg virus VP40 dimer (b) The interactive 3D viewer illustrating selected binding modes (c) Residue wise interaction between ligand and protein obtained through Discovery Studio.

The docked complex of Marburg virus VP40 dimer with Gliotoxin is shown in Fig 7. It was observed that Gliotoxin made six hydrogen bonds with Gln143, Arg136, Pro134, Pro298, Ile88, and Gln276. It was also involved in hydrophobic interactions with Pro85 and Pro202.

**Fig 8:**
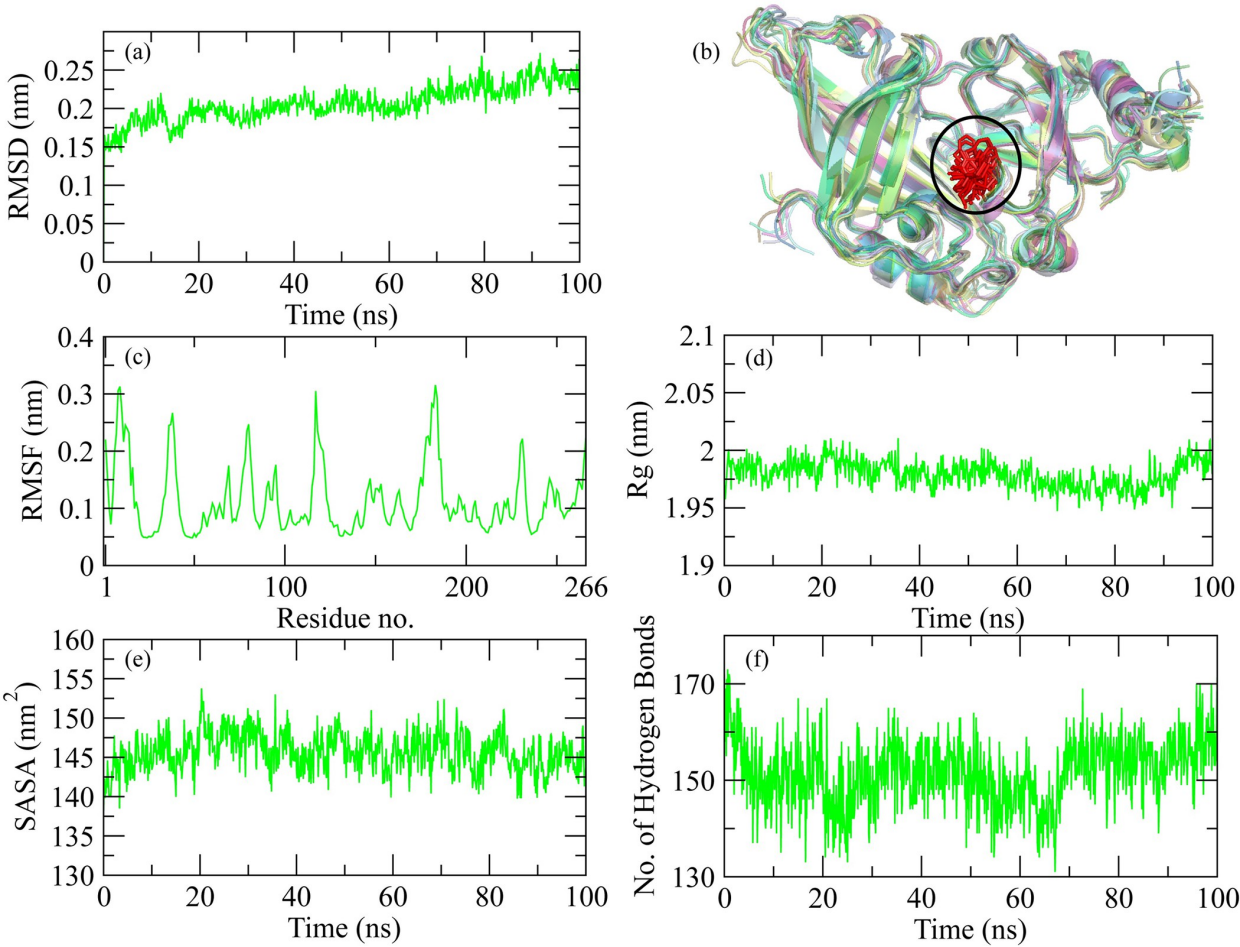
Graphical representation of Marburg virus VP40 dimer with Gliotoxin over a 100ns MD simulations. (a) RMSD graph showing the interaction between protein ligands. (b) Alignment of the snapshots extracted from MD trajectory. (c) RMSF showing the fluctuation in protein ligand complex on different amino acid residues plotted on x-axis. (d) Radius of gyration showing the gain of compactness of complex towards the end of simulation. (e) SASA value indicating the stable protein ligand interaction. (f) Number of hydrogen bonds affecting the interactions of protein ligand complex.

**MD simulation of Marburg virus VP40 dimer and Gliotoxin complex.** The RMSD score is a measure of how much the configuration of atoms in a protein has varied over time. Lower RMSD value is an indication of rather stable interaction. The RMSD analysis of Marburg virus VP40 dimer with Gliotoxin below demonstrated a gradual increase in RMSD value which refers to the decreasing stability of the complex over a 100ns simulation (Fig 8A). The alignment of extracted snapshots revealed the stability of protein-ligand complex during simulation (Fig 8B). RMSF is a measure of protein flexibility and an inconsistency with sudden increase and decrease in RMSF value can be seen throughout. At certain residues, 54, 76, 117, 220, and 268 the complex showed exceptionally high flexibility (Fig 8C). Radius of gyration defines the behavior of protein in terms of compactness. Though a highly fluctuating or abrupt trend in graph of Rg indicates that every second the complex went through a series of folding and unfolding events of the chains to survive, overall, there is an increase in Rg value from start till end (Fig 8D). SASA and number of hydrogen bond analysis showed that a highly variable trend was observed throughout which refers to a continuously changing availability of nearby interacting solvent molecules and hydrogen bond formation and breakage every ns respectively (Fig 8E and 8F). From this variability in trend, we can infer that the complex hasn’t significantly affected the SASA and H-bond formation parameter.

### MMPBSA of Marburg virus VP40 dimer and Gliotoxin complex

**Fig 9:**
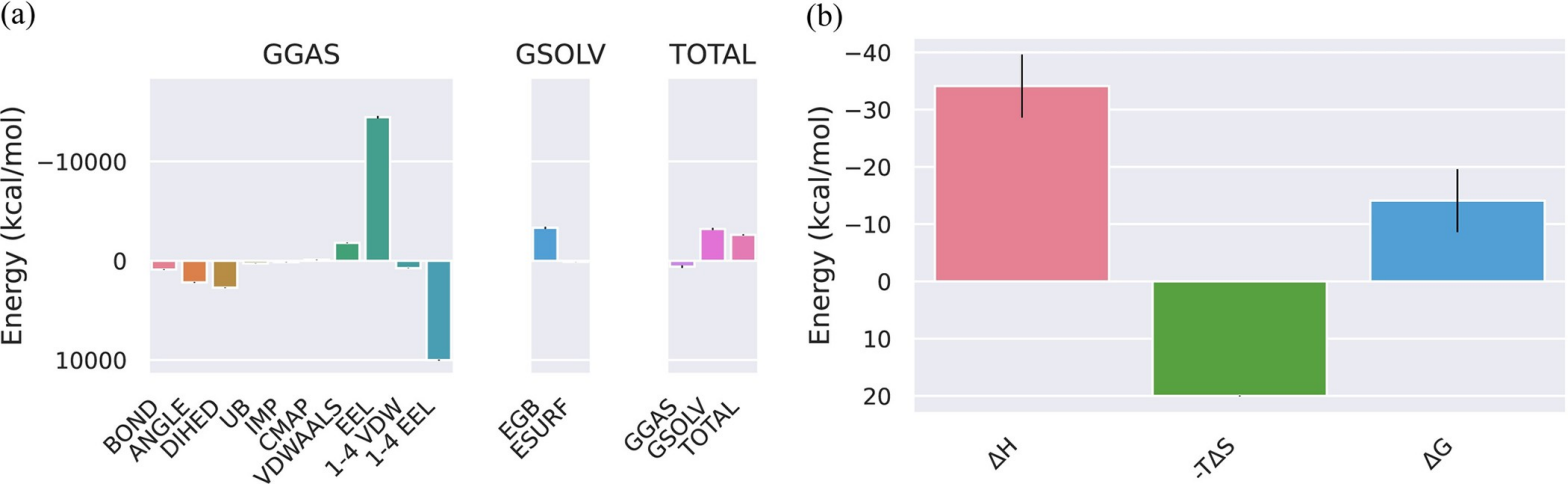
(a) The negative binding energy for the Marburg virus VP40 dimer and Gliotoxin complex represents the stability in the structure of the protein after the attachment of Gliotoxin ligand. (b) The decrease in the enthalpy makes the complex stable while the entropy is showing an unfavorable interaction protein between the protein and the ligand Gliotoxin.

The different phases of Marburg virus VP40 dimer and the change in the bonds and the energy that takes place in it because of the Marburg virus VP40 dimer binding with Gliotoxin is represented in Fig 9(A). The total energy of the protein and ligand complex decreases for both GGAS and GSOLV which shows the stability in the structure of the Marburg virus VP40 dimer after the attachment of the ligand Gliotoxin. The results of the binding energy of the Marburg virus VP40 dimer protein_ Gliotoxin complex are represented in Fig 9(B). These results are computed from the GB and IE methods which are involved in the standard MM/GBSA calculations. A detailed analysis showed the enthalpic component (ΔH) as favorable (negative value) to the binding process. At the same time, the entropic term (−TΔS) has given an unfavorable (positive value) of the energy. The Gibbs free energy (ΔG) has shown a decrease in the energy of the complex formation which explains the stability of the complex.

### 4. Ebola VP35 Interferon Inhibitory Domain and Ozazino-cyclo-(2,3-dihydroxyl-trp-tyr)

**Fig 10:**
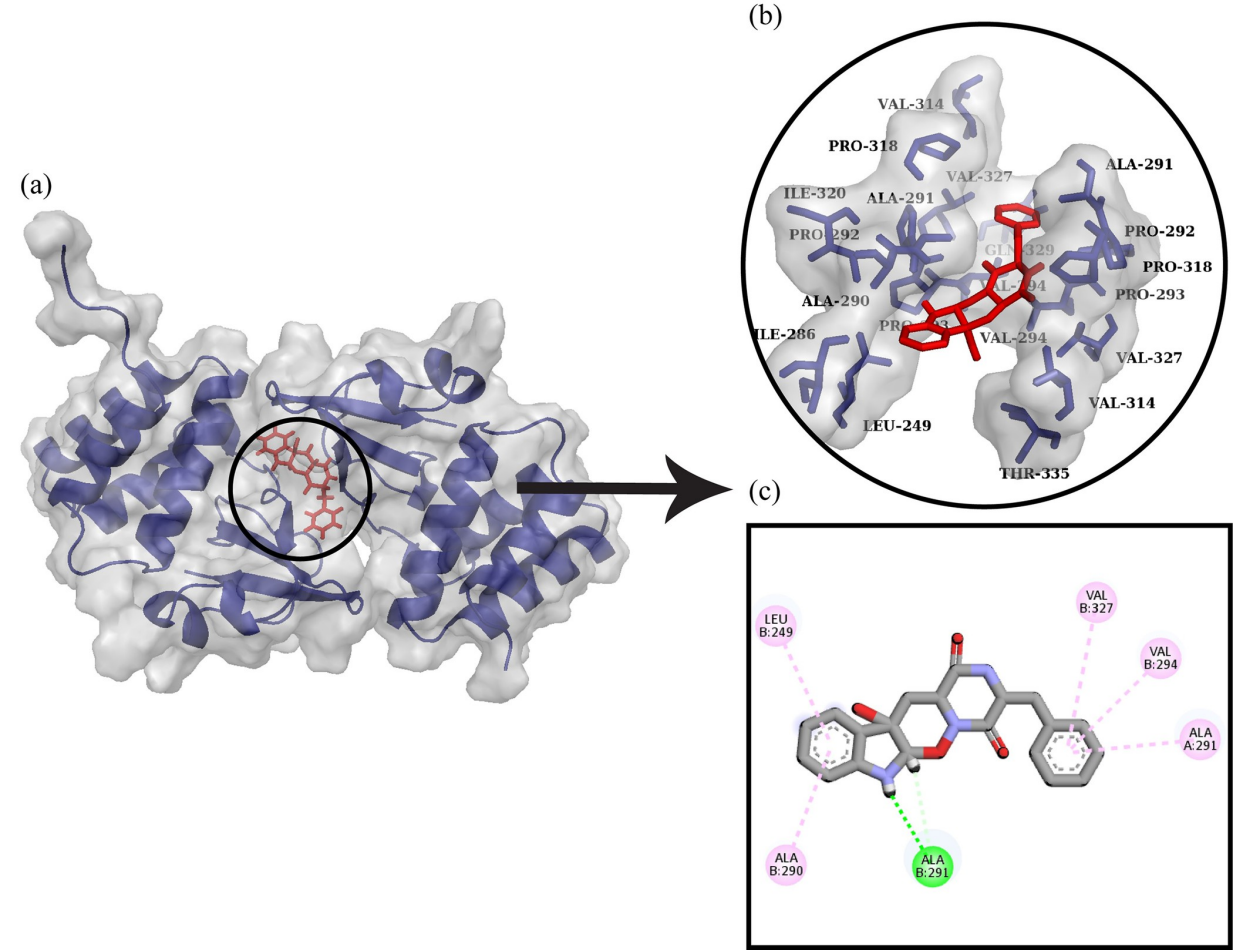
Protein-ligand docking. (a) The ligand Ozazino-cyclo-(2,3-dihydroxyl-trp-tyr) is docked with Ebola VP35 Interferon Inhibitory Domain. (b) The interactive 3D viewer illustrating selected binding modes (c) Residue wise interaction between ligand and protein obtained through Discovery Studio.

The docked complex of Ebola VP35 Interferon Inhibitory Domain and Ozazino-cyclo-(2,3-dihydroxyl-trp-tyr) is shown in Fig 10. It was observed that ligand formed one hydrogen bond with Ala291 and five alkyl interactions with Ala290, Leu249, Val327, Val294, and Ala291.

### MD simulation of Ebola VP35 Interferon Inhibitory Domain and Ozazino-cyclo-(2,3-dihydroxyl-trp-tyr) complex

**Fig 11:**
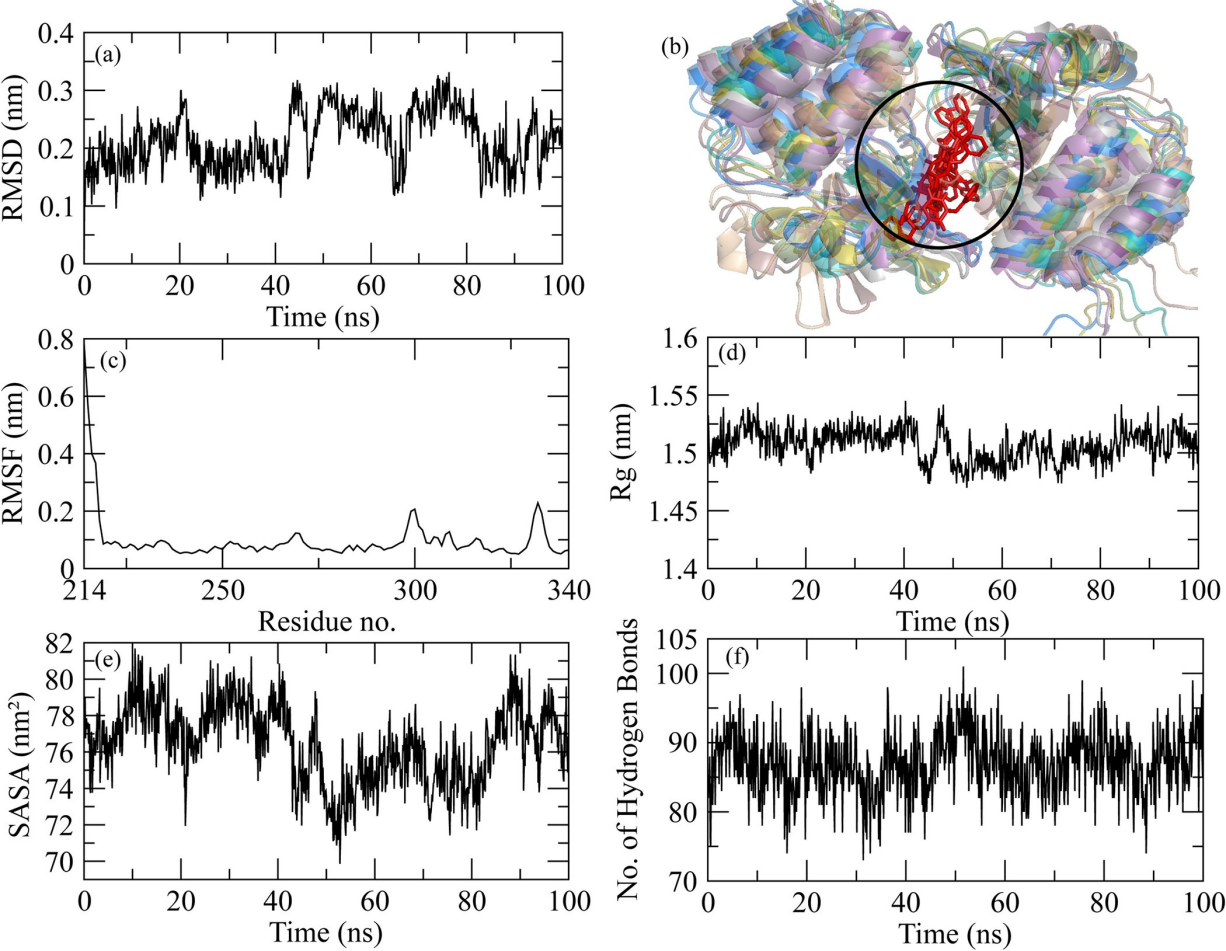
Graphical representation of molecular dynamic simulation at 100ns for Ebola VP35 Interferon Inhibitory Domain and Ozazino-cyclo-(2,3-dihydroxyl-trp-tyr). (a) RMSD for the stability of the respective protein-ligand complex. (b) Alignment of the snapshots extracted from MD trajectory. (c) RMSF explains the flexibility of the protein as per the fluctuation of the amino acid. (d) The Radius of Gyration shows the compactness in the protein as the ligands bind to it. (e) The accessibility of the solvents for protein can be predicted through the SASA value. (f) The breaking and formation of the hydrogen bonds with the passage of time is represented graphically.

The molecular dynamic simulation analysis for Ebola VP35 Interferon Inhibitory Domain and Ozazino-cyclo-(2,3-dihydroxyl-trp-tyr) protein-ligand complex is represented graphically. The RMSD of the protein after the binding of ligand is represented in Fig 11A. The RMSD explains the deviation of the protein from its standard which gives us information about the stability of the protein. After the binding of ligand to the protein, it was observed that the RMSD value started increasing from the 0ns, which shows the less stable or weaker interaction between the protein and ligand over time. While the snapshots alignment revealed the stable formation of complex during simulation (Fig 11B). RMSF for the following complex is shown in Fig 11C. The RMSF describes the flexibility of a protein after its interaction with the ligand. The graph shows the decrease in the RMSF value which shows the decreased flexibility of protein after the binding of the ligand. The radius of gyration explains the compactness in the structure of the protein that after the binding of ligand either the compactness of the protein increases or decreases. The decrease in the Rg value was observed at 45ns (Fig 11D) which explains the binding of ligand has caused a conformational change resulting in folding of the protein. SASA for the protein-ligand complex is represented in Fig 11E. The SASA value describes the availability of the surface molecules in the surrounding of protein which helps in its interaction with the ligand. At the start, there was an increase in the SASA value, but it starts to decrease from 50ns which shows that the accessibility of solvent to the protein decreases as the ligand binds to it. The number of hydrogen bonds (Fig 11F) graphs showed that the binding of ligand to protein hasn’t significantly affected the trend of H-bond formation.

### MMPBSA of Ebola VP35 Interferon Inhibitory Domain and Ozazino-cyclo-(2,3-dihydroxyl-trp-tyr) complex

**Fig 12:**
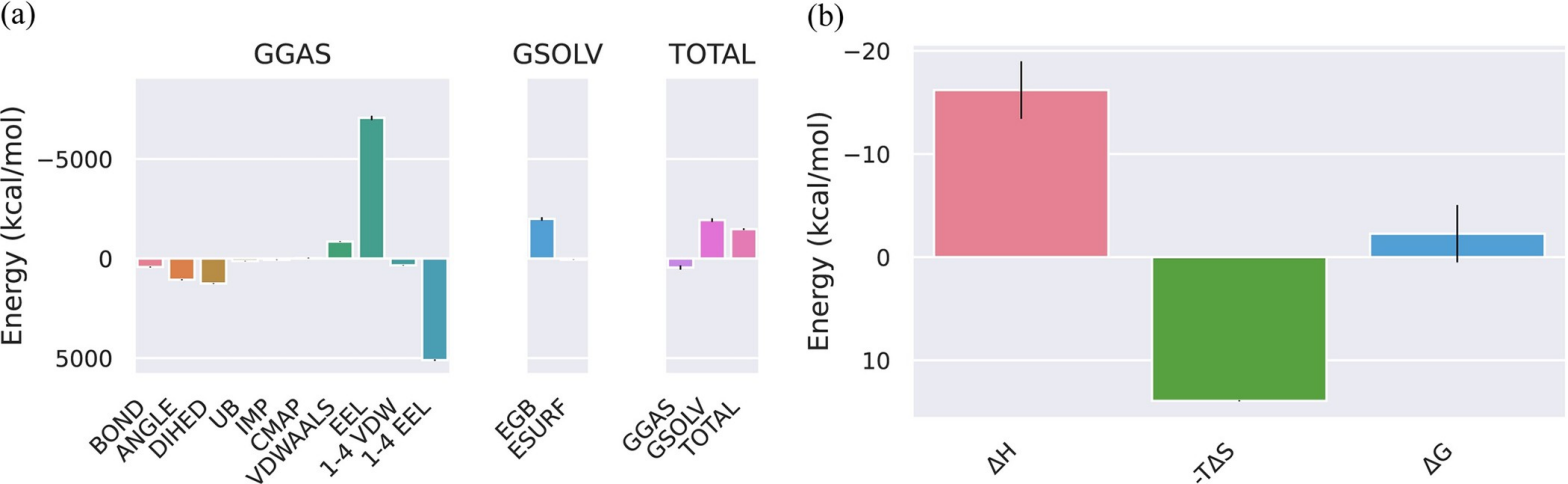
(a) The total energies of GGAS and GSOLV of the Ebola VP35 Interferon Inhibitory Domain-Ozazino-cyclo-(2,3-dihydroxyl-trp-tyr) complex were calculated by the GB method. (b) The instability in the structure of the following protein-ligand complex has been represented from the entropy values.

The energetics of the Ebola VP35 Interferon Inhibitory Domain to the ligand Ozazino-cyclo-(2,3-dihydroxyl-trp-tyr) is represented in Fig 12. The different phases of the following protein and the change in the bonds and the energy that takes place in it because of the binding of the ligand to the protein is represented in Fig 12(A). The total energy of the protein and ligand complex decreases for both GGAS and GSOLV which shows the stability in the structure of the protein after the attachment of the Ozazino-cyclo-(2,3-dihydroxyl-trp-tyr) ligand. The results of the binding energy of the Ebola VP35 Interferon Inhibitory Domain-Ozazino-cyclo-(2,3-dihydroxyl-trp-tyr) complex are represented in Fig 12(B). These results are computed from the GB and IE methods involved in the standard MM/GBSA calculations. A detailed analysis of the contributions showed the enthalpy component (ΔH) as favorable (negative value) to the binding process. At the same time, the entropic term (−TΔS) has given an unfavorable (positive value) of the energy. The Gibbs free energy (ΔG) has shown a decrease in the energy of the Ebola VP35 Interferon Inhibitory Domain-Ozazino-cyclo-(2,3-dihydroxyl-trp-tyr) complex formation which explains the stability of the complex.

### Toxicity analysis

The toxicity profiles of the compounds were predicted by ProTox-II server. The insilico toxicity prediction of the compounds reduces the cost of experimentation. The toxicity properties such as Hepatotoxicity, Carcinogenicity, Mutagenicity, and Cytotoxicity were predicted and compared. It can be observed that all the compounds were inactive for Hepatotoxicity, Carcinogenicity, Mutagenicity, and Cytotoxicity (Table 3).

**Table 3:** The toxicity profiles of the identified compounds.

Compounds	Hepatotoxicity	Carcinogenicity	Mutagenicity	Cytotoxicity
Dehydroaustinol	Inactive	Inactive	Inactive	Inactive
Austinol	Inactive	Inactive	Inactive	Inactive
Gliotoxin	Active	Inactive	Inactive	Inactive
Ozazino-cyclo-(2,3-dihydroxyl-trp-tyr)	Inactive	Inactive	Inactive	Inactive

## Discussion

Drug discovery approaches such as virtual screening and molecular docking have triggered a dramatic shift in the profession by speeding up and streamlining the process of identifying potential therapeutic compounds. Virtual screening uses the capacity of computational simulations to rapidly sift through enormous databases of chemical compounds, estimating their binding affinities and interactions with disease-related target proteins. This method considerably speeds up the discovery of lead compounds by eliminating the requirement for time-consuming and expensive large-scale experimental studies [22, 23]. A key component of virtual screening is molecular docking, which mimics the binding process between a small molecule and a target protein, allowing the structure and stability of their complex to be predicted. This allows researchers to rank and prioritize candidate compounds based on their binding energies, perhaps highlighting those with the greatest likelihood of exhibiting the desired therapeutic effect [24]. Virtual screening and molecular docking have not only reduced the drug discovery timeline from years to months by combining advanced algorithms, structural biology insights, and high-performance computing, but they have also democratized access to computational resources, allowing researchers worldwide to contribute to the development of novel drugs and treatments [25, 26]. Different studies have used these techniques to identify novel inhibitors [27–30]. Hence, these techniques play an important role in identifying inhibitors.

In this study, the protein structures of Marburg virus VP40 Dimer, matrix protein VP40 from Ebola virus Sudan, Ebola VP35 Interferon Inhibitory Domain, and VP35 from Marburg virus were retrieved from Protein Data Bank. The structural and functional properties of Gliotoxin, Austinol, Ozazino-cyclo-(2,3-dihydroxyl-trp-tyr), Dehydroaustinol compounds were gathered using the PubChem database. Numerous inhibitors have been proposed against these proteins, but our study consists of inhibitors against both viruses [31–33]. Significant inhibitors or lead compounds can be identified from MCULE, a web-based library with millions of potentially synthesized compounds using a technique called structure-based virtual screening. More than 5,000,000 different ligands were employed in this investigation to investigate the CMAH active site. The molecular docking of CMAH with the naturally occurring ligand was carried out using the specified grid box. Vina integration was used in the software for digital screening. After evaluating the inhibitors’ binding affinities with the Vina-docking score, the two strongest inhibitors were chosen. Furthermore, AdmetSAR was utilized to investigate the pharmacokinetics of the inhibitors chosen. The pharmacokinetic features of inhibitors, typically referred to by the acronym ADME (Absorption, Distribution, Metabolism, and Excretion), are critical in establishing the effectiveness, safety, and general acceptability of these substances for use as drugs. Predicting ADME features of inhibitors is crucial for drug development since it allows possible difficulties to be identified early in the process. This information assists researchers in identifying the most promising compounds for further research, refining their structures to improve ADME profiles, and avoiding candidates with unfavorable properties that could lead to poor clinical outcomes or safety concerns [34].

CB Dock was used to carry out protein-ligand docking. Protein-ligand interactions usually involve receptor flexibility, which enables selectivity in ligand recognition. Gliotoxin was used as a ligand with Marburg virus VP40 Dimer, Austinol with matrix protein VP40 from Ebola virus Sudan, Ozazino-cyclo-(2,3-dihydroxyl-trp-tyr) with Ebola VP35 Interferon Inhibitory Domain, and Dehydroaustinol with VP35 from Marburg virus. All ligands bind favorably to their target proteins.

Docked complexes were further evaluated through MD simulation and MMGBSA/MMPBSA analysis. MD allows for the building of an ensemble of structures that may be used to compute thermodynamic potentials such as binding free energy with great precision [35, 36]. MD simulation and MMPBSA studies revealed that these compounds were stable as potent inhibitors within the proteins binding pocket. These inhibitors may give rise to a therapeutic solution by efficiently inhibiting and targeting the catalytic function of their respective target proteins. Hence, our findings regarding the bioactivity of Manzamine A N-oxide, Isonaamine E, 32,33-dihydro-31-hydroxymanzamine A, 32,33-dihydro-31-hydroxymanzamine A, and 8-Hydroxymanzamine A warrant additional research for structure-based lead optimization.

### Conclusion

The current study aimed to conduct molecular docking and virtual screening on deep-sea fungal metabolites targeting VP30 and VP40 of Ebola and Marburg viruses to design therapeutic interventions. The docking experiments were also validated by molecular dynamics simulations and the MM-PBSA binding free energy. However, further studies, including experimental validation, are necessary to confirm these findings.
